# Designing and validating an adaptation tool for endometriosis: an exploratory mixed method study protocol

**DOI:** 10.1186/s12978-025-01948-9

**Published:** 2025-01-26

**Authors:** Sanaz Mollazadeh, Nahid Marvi, Talat Khadivzadeh

**Affiliations:** 1 Reproductive Health, Nursing and Midwifery Care Research Center, Midwifery Group, Mashhad University of Medical Sciences, Mashhad, Iran; 2 Reproductive Health, Department of Midwifery, School of Nursing and Midwifery, Mashhad University of Medical Sciences, Mashhad, Iran

**Keywords:** Endometriosis, Roy adaptation model, Qualitative study

## Abstract

**Background:**

Endometriosis is a benign and chronic gynecological estrogen-dependent condition. Research findings have highlighted its impact on different aspects of women's lives. Enhancing quality of life and supporting the well-being of those affected is advised. Yet, none of the conducted studies have taken action toward these objectives. Therefore, the present study will be conducted with the aim of "Designing adaptation tool for endometriosis".

**Methods/design:**

The method used in this study is an exploratory mixed-method study. The study will consist of two phases, starting with a qualitative phase followed by a quantitative phase. Upon approval Ethics Code from the endometriosis clinic at Imam Reza Hospital in Mashhad, Iran. The research will involve women of reproductive age diagnosed with endometriosis. In-depth and semi-structured interviews with open-ended questions will be conducted. The research aims to explore the experiences of women with endometriosis in adapting to the condition, utilizing qualitative content analysis with an approach based on the "ROY adaptation model." Sampling will be purposive until data saturation is achieved. Data analysis will follow the suggested steps using Elo Kingas' method and MAXQDA20 software. Tool design will involve an inductive approach (informed by qualitative findings) and a comparative method (based on literature review) to develop and refine tool items.

**Discussion:**

This study is the first to employ a mixed-method approach in developing an adaptation tool for endometriosis. It uncovers underlying issues in the attitudes of patients, medical staff, and healthcare providers, offering insight into factors that can enhance the health and quality of life of affected women. The research findings can inform the creation of a relevant strategy for policymakers, planners, and healthcare professionals to better address the needs of women impacted by endometriosis.

*Ethical code*: IR.MUMS.NURSE.REC.1403.069.

## Plain language summary

Endometriosis is a benign and chronic gynecological estrogen-dependent condition characterized by the presence of endometrial tissue outside the uterus, leading to a persistent inflammatory response in the pelvic region. Endometriosis lacks a definitive cure, and research has highlighted its profound impact on various aspects of women's lives, including physical health, mental well-being, social interactions, sexual and marital relationships, economic stability, employment status, educational pursuits, and lifestyle choices. Given the absence of a definitive treatment for endometriosis, it is vital to address the concerns surrounding the condition to enhance the overall well-being of affected individuals. Providing support and understanding to women dealing with endometriosis is a crucial and beneficial approach. By exploring women's perspectives, experiences, and cultural expectations related to endometriosis, valuable insights can be gained to develop tailored adaptation strategies and tools aimed at enhancing the quality of life for those affected by the condition.

## Background

Endometriosis affects 2–10% of women in reproductive age and is characterized by the presence of ectopic endometrial tissue, leading to chronic inflammatory reaction. The ovary, uterosacral ligament, Douglas, cervix, sigmoid colon, and pelvic peritoneum are the most common sites for replacement in the pelvic cavity [1–3]. Endometriosis can be asymptomatic in some patients and may be incidentally discovered [4]. Women with endometriosis often experience symptoms such as infertility, periodic and non-periodic abdominal pain, painful menstruation, bloating, diarrhea or constipation, painful intercourse, painful urination, and painful defecation [5]. There is no definitive cure for endometriosis, and women with this chronic, estrogen-dependent disease may experience a variety of symptoms, from mild pain to severe debilitation [6]. The etiology of endometriosis is intricate and multifactorial [7]. While the exact cause of endometriosis remains unclear, retrograde menstruation is widely recognized as a major contributing factor [8, 9]. Qualitative studies examining the impact of endometriosis on women's lives have revealed extensive effects across various dimensions, including physical health, psychological well-being, social interactions, sexual and marital relationships, economic factors, employment, education, and lifestyle [1, 6, 10]. Given that endometriosis is a chronic condition with no conclusive cure and has a major impact on women's lives [11], it is crucial to address adaption habits and endometriosis-related issues to disseminate valuable knowledge that could enhance the quality of life for affected women [12]. Regrettably, endometriosis is a deeply troubling ordeal and women's issues often go undetected, resulting in the long-term effects of the condition being overlooked and causing enduring distress [13]. Therefore, embracing an adaption lifestyle appears to be a beneficial and crucial strategy for women dealing with endometriosis [4, 5, 14].

### Study aim

By analyzing the findings of this study, we can gain insights into women's perceptions and experiences of endometriosis, delving into cultural nuances and expectations. Ultimately, this can lead to the development of an adaptation tool to enhance the quality of life for women affected by endometriosis.

### Specific objectives

**The main objective:**

Designing of the endometriosis adaptation assessment questionnaire.

**The main research question:**

What are the dimensions of an adaptation tool in women with endometriosis?

**The goal of the qualitative phase:**

Explaining women's understanding and experience of adaptation to endometriosis.

**The specific objectives of the quality stage:**

Explaining women's experience of adaptation to endometriosis.

Explaining the dimensions of adaptation with endometriosis.

**The objective of the quantitative stage:**

Designing of the endometriosis adaptation assessment tool.

**Specific objectives of the quantitative phase:**

Explaining the items of the endometriosis adaptation tool.

### Practical purposes

Using this tool, it will be possible to assess adaptation to endometriosis in women and identify those requiring intervention. By measuring adaptation with this tool, interventions can be targeted for women in need, evaluating the success and efficacy of the interventions to aid in the adaptation process for effected women with endometriosis. Subsequently, a support program can be developed based on these findings. Implementing this program within the healthcare system by staff and service providers can facilitate positive adaptation for women affected with endometriosis.

## Methods/design

### Study design

The study will employ a mixed-method approach. It will begin with an exploratory phase utilizing qualitative methods, followed by a quantitative phase [15].

### Sampling

The present study was confirmed by the Ethics Council of Mashhad University of Medical Sciences (ethics code: IR.MUMS.NURSE.REC.1403.069). Subsequently, data collection will commence at the endometriosis clinic of Imam Reza Hospital in Mashhad. The researcher will attend the endometriosis clinic, eligible women will be identified by referring to their medical records and will be invited to participate in the study using random sampling and considering the inclusion and exclusion criteria. Before the study, written informed consent will be obtained from the participants. The researcher will introduce herself to the patients and will provide them with necessary explanations regarding the study's purposes. Then patients will be given written questionnaires to fulfill.

### Inclusion criteria

Women aged 15–49 years with a diagnosis of endometriosis through an open surgery, laparoscopy, histological diagnosis of endometriosis, the presence of endometrioma cyst, or diagnosis by ultrasound and MRI confirmed by a gynecologist. Moreover, those women diagnosed with endometriosis localized in the pelvis and peritoneum, experienced the onset of endometriosis symptoms at least one year, diagnosed with no presence of endometriosis in the partial region or remote organs, (e.g. lungs and brain), had Iranian nationality, be married, be literate to answer questions, be non-menopausal status (amenorrhea for over a year), did not suffer from any other major diseases, such as mental disorders, severe depression, schizophrenia or chronic diseases such as diabetes, kidney disease, rheumatological disorders, cancer, and life-threatening diseases will be involved in the study.

Contrary to the results of some studies suggesting no relationship between the severity or stage of endometriosis and the severity of the symptoms [16–19], we will not consider the stage of the disease as an inclusion criterion in this study.

### Exclusion criteria

The women who decline to further cooperate and submit an incomplete questionnaire (left unanswered more than 10 percent of the questions) will be excluded from our study.

### The first phase: qualitative study

The first phase consists of an exploratory qualitative study with directed analysis. This qualitative method will be used to explore and explain the understanding and experience of the effects of the disease and adaptation in women with endometriosis.

### Sampling method

A directed qualitative content analysis approach will be adopted in this study. The COREQ checklist will be used for reporting the results of this study.

### Data collection

At this stage, using semi-structured interviews with targeted sampling method and with maximum diversity in terms of age, education, occupation, degree of the endometriosis, fertility or infertility. The women will be selected and their experiences and feelings will be examined and described using the guide of questions based on theory. After selecting the women and contacting them in person or by phone, an interview will be arranged. The interview will be conducted in a quiet room with the presence of the researcher and the participant at the place agreed between the researcher and the participant, and the participant's voice will be recorded during the interview with their consent.

### Data analysis

Data analysis starts simultaneously with data collection [20]. For data analysis, Elo and Kingas [21] comparative analysis will be used, which consists of three stages: preparation, organization and reporting. This method is used when the analysis structure is based on previous knowledge [21]. The two-part preparation stage includes choosing the unit of analysis and finding the logical connection of the data with the subject as a whole. Which starts with the selection of the unit of analysis and then goes on to understand the logical connection between the data and the subject as a whole according to the research question. We will use of four criteria to evaluate the accuracy of the qualitative data (Credibility, Dependability, Confirmability, Transferability [22]. The interview texts and codes will be organized in MAXQDA 20.

A- Selecting the unit of analysis: In this step, the text of the interview will be implemented and the implemented text is considered as the unit of analysis. Both the obvious content, i.e. the transcript of the interview, and the hidden content, including silence, pauses, sighs, laughter, and body posture, will be also considered.

### The second phase of the study (quantitative phase)

In the first step of the quantitative phase, the four-step method of Waltz et al. will be used to make the tool, which includes 1-choosing a conceptual model, 2-determining measurement objectives, 3-determining the plan and map, and 4-making tools [23]. In the first step in this study, the adaptation model will be selected as a conceptual model to measure the structure of adaptation to endometriosis. In the second step, the tool should be able to measure the degree of adaptation with endometriosis. In the third stage, by using the results of the first phase, the areas and dimensions of adaptation that can be measured, as well as the determination of the primary items, will be carried out. In the last stage, appropriate items will be designed based on the operational definitions extracted from the content analysis and available resources. The items will be initially determined, then a scale will be added to them for measurement. In such a way that the information obtained from the interviews will be summarized, based on this information and using the available sources, a repository of items will be formed. From this repository, the initial items of the endometriosis adaptation questionnaire will be selected. Then, in the next step, the way to answer the questions of the questionnaire and determine the way to score these questions will be determined. The primary tool will be checked by the research team and simple and understandable expressions will be used. After revising the tool, the face validity of the tool will be checked.

**Face validity**: only examines the degree of conformity of the instrument's appearance with the subject it claims to measure, and this work will be done by the target group. Two qualitative and quantitative methods will be used for face validity. In qualitative face validity, several women from the target group (women who have endometriosis) will be interviewed face to face, and the level of difficulty (phrases and words that are difficult for the respondents to understand), the degree of appropriateness (property and appropriate communication) The items will be checked with the purpose, scale and dimensions of the questionnaire) and the ambiguity of the items (misconceptions of phrases and items). In quantitative formal validity of the obtained questionnaire, after modifying the problematic items based on the opinion of the participants, in order to determine the importance of each item and remove inappropriate items, it will be given to a limited population of women who have endometriosis. Answers will be determined as completely important, somewhat important, moderately important, slightly important, and not important at all. Answers will be scored on a 5-point Likert scale. Using the item impression, the importance of each item will be determined.

The **content validity** will be checked with two quantitative and qualitative methods. In the qualitative method, the judgment of experts in the fields of midwifery, reproductive health, nursing, and women will be used and they will asked to give their opinion about the tool by studying it carefully and the tool in terms of grammar, The use of appropriate words, placement of items in their proper place, and appropriate scoring are checked by experts (59) and after that, a quantitative content validity check will be performed, during which two indexes of content validity ratio (CVR) and content validity index (CVI) will be calculated [24–26]. The content validity index examines the necessity of an item from the point of view of experts. In order to determine CVR, a number of experts will be asked to review the items based on three scales: necessary, useful but not necessary, and not necessary. The answers will be calculated based on the CVR formula. The minimum validity ratio index will be determined from the Lawshe table based on the number of responding experts, and the items with lower CVR will be removed. In this way, inappropriate items will be corrected. The CVI analysis will be based on Waltz's and Basel's reliability index. The questionnaire will be given to a number of experts and they will be asked to determine the degree of relevance of the items based on a four-part Likert scale (not relevant, relatively relevant, relevant, and completely relevant). The CVI score will be obtained by dividing the number of experts who have chosen the 3rd and 4th rank by the total number of responding experts. The content validity of the entire instrument (S-CVI) will be calculated by calculating the validity index for each item (I-CVI) and then the average of the total I-CVI in all items, that is, the S-CVI/Avg approach will be used. The acceptance criteria is 0.90 and above [26–28]. The kappa statistic is an agreement index that is matched by chance. Adjusted kappa value greater than 0.74 is considered excellent, between 0.6 and 0.74 is considered good, and less than 0.6 is considered poor [29].

**Construct validity**: exploratory factor analysis and confirmatory factor analysis will be used to determine construct validity. The purpose of exploratory factor analysis is to find and specify relationships between variables and it is used to discover classes of variables that are most related to each other. To determine the validity of the construct in hospitals, it is used with available sampling method. According to the number of items, the sample size is determined (3–5 women per item). The samples will be selected from Iranian women living in Mashhad who have a history of endometriosis. To check the adequacy of the sample for factor analysis and the ability to classify the data, the Keyser-Meyer-Elkin sampling index test and the Bartlett test will be used. It is a gravel graph and a varimax epoch. Expressions with a factor load of less than 0.4 will be removed. KMO greater than 0.5 and the significance of Bartlett's test (P < 0.05) indicate the adequacy of the sample for factor analysis. Confirmatory factor analysis will be done with the aim of determining whether the relationships between the variables in the assumed model are similar to the relationships between the variables in the data. And it determines the agreement of the assumed covariance with the observed covariance. It is done in 5 steps including determining the model, identifying, estimating the model parameters, evaluating the fit of the model with the data, and modifying or re-determining the model to improve the fit. Chi-square statistic is used to determine the difference between assumed and observed relationships, which is more than 0.05 desirable. Due to the sensitivity of chi-square to the sample size, the ratio of chi-square to degrees of freedom is used. NFI, GFI, AGFI, CFI, and RMSEA indices are used. The prerequisite for performing factor analysis is to confirm the normality of the data [28]. The resulting data will be analyzed with the help of Lisrel software.

**Reliability:** In order to determine the reliability of the tool, the reliability of the stability and the uniformity of the tool will be used. To determine internal reliability, Cronbach's alpha coefficient will be used in completed questionnaires. Alpha coefficient above 0.7 is acceptable. To evaluate the stability of the instrument, test–retest will be used in a limited number of participants. The scores obtained in two stages will be compared using the intra-class correlation index test. By using the standard error of measurement, the agreement of the instrument will be checked and the range of changes of the obtained scores will be determined. In the present study, SEM agreement will be used.

### Research method: sequential exploratory combination

The second phase of the study, which is tool building, will be determine and measure face validity, content validity, construct validity, and tool reliability.

**The statistical method** will be used to describe or compare the main measurable outcomes and the outcome used to calculate the sample size:

Cronbach's alpha coefficient to determine the internal consistency of the instrument, interclass correlation test to determine the stability of the instrument, exploratory factor analysis to discover and determine the classes and relationships between instrument variables, Keyser-Meyer-Elkin test, Bartlett's test, Varimax cycle, sand graph to determine the adequacy of the sample in exploratory factor analysis and confirmatory factor analysis, to determine the similarity of the relationships between the variables in the assumed model with the relationships between the variables in the data and to determine the agreement of the assumed covariance with the observed covariance, RMSE, CFI will be used., AGFI, GFI, NFI will be used and descriptive statistical tests will be used to compare the demographic characteristics of the participants. Prevalence, possible effect of the intervention or… along with the dispersion of the main outcome to calculate the sample size along with its assumption (from a source, pilot study, consensus of researchers, minimum valuable clinical effect or…):

The final sample size with reference to the amount of type 1 and type 2 error tolerated (in total and in each research group along with the possible time to reach it.

This study is the first to develop a tool for assessing adaptation to Endometriosis, introducing the Endometriosis Adaptation Scale. Endometriosis has a significant impact on the quality of life and daily activities of affected women, including interpersonal relationships and sexual desires, as well as the ability to do daily tasks, work and fertility, interruptions in education and work, and reduced social participation, and physical and mental wellbeing [30–32]. Unfortunately, even though life with endometriosis is very difficult for many patients, the problems of these patients have not been given much attention and women suffer from the harmful effects of this disease for a long time. Patients with endometriosis face a world full of false information about their disease, taboos, and unanswered questions, lack of timely diagnosis, and problematic treatments, which are covered with a painful, stubborn, and chronic disease [30, 31].

This instrument was formulated from a qualitative study following the Roy Adaptation model, supplemented by items from other adaptation and coping tools. Adapting to this crisis necessitates mental and physical preparation, as well as social and familial support [33]. Adaptation is defined as the alignment between individual needs and external demands [34]. In evolutionary theory, adaptation refers to essential structural or behavioral changes that sustain life [35]. The physiological, social, and psychological aspects of adaptation are interdependent, meaning that changes in one area affect the others [36]. Adaptation is a complex, multidimensional, subjective, and interactive concept influenced by various factors and shaped by societal context [37]. Currently, there is no consensus on the definition and indicators of adaptation [38]. Adapting to stressors is a continuous, progressive interaction between an individual and their environment [39]. Understanding the effects of stress and adaptation is crucial for enhancing health outcomes [40]. One widely used model for adapting to diseases and stress is the Zinc Adaptation Model from 1970, which posits that adaptation depends on the nature of the stimulus and an individual's response [41]. It categorizes stimuli into main, background, and residual types, to which individuals provide adaptive or ineffective responses. The model also distinguishes four dimensions: self-concept (psychological), social interactions (playing), and emotional aspects (dependence and independence) as key components of adaptation [42]. This study leverages the Roy Adaptation model for its detailed examination of patients through interviews, observations, and measurements, focusing on ineffective behaviors and their stimuli in relation to the disease. Ultimately, the study aims to elucidate women's experiences of adapting to Endometriosis and establish the psychometric properties of a measurement tool for this adaptation.

## Data Availability

No datasets were generated or analysed during the current study.

## References

[1] Masoumeh Namazi ZBM, Zareiyan A, Jafarabadi M. Exploring the impact of endometriosis on women’s lives: a qualitative study in Iran. 2020. 10.1002/nop2.744 PMC8046092 33354928

[2] Parazzini F, et al. Epidemiology of endometriosis and its comorbidities. Eur J Obstet Gynecol Reprod Biol. 2017;209:3–7. 27216973 10.1016/j.ejogrb.2016.04.021

[3] Zondervan KT, Becker CM, Missmer SA. Endometriosis. N Engl J Med. 2020;382:1244–56. 32212520 10.1056/NEJMra1810764

[4] Ghonemy GE, El Sharkawy NB. Impact of changing lifestyle on endometriosis related pain. IOSR J Nurs Health Sci. 2017;6(2):120–9.

[5] Abd-Elaziz Ibrahim N, et al. Effect of application of health promotion model on lifestyle of women with endometriosis. J Nurs Sci Benha Univ. 2021;2(2):154–69.

[6] Moradi M, et al. Impact of endometriosis on women’s lives: a qualitative study. BMC Womens Health. 2014;14(1):1–12. 25280500 10.1186/1472-6874-14-123 PMC4287196

[7] Mollazadeh S, et al. Association between health behaviours during menstruation and endometriosis. J Clin Diagn Res. 2019;13(2).

[8] Králíčková M, et al. Endometriosis and risk of ovarian cancer: what do we know? Arch Gynecol Obstet. 2020;301(1):1–10. 31745637 10.1007/s00404-019-05358-8

[9] Kvaskoff M, et al. Endometriosis: a high-risk population for major chronic diseases? Hum Reprod Update. 2015;21(4):500–16. 25765863 10.1093/humupd/dmv013 PMC4463000

[10] Riazi H, Tehranian N, Ziaei S, Mohammadi E, Hajizadeh E. Perception and experiences of patients with endometriosis about pain: a qualitative study. J Mazandaran Univ Med Sci. 2015;25(129):57 (Persian).

[11] Mollazadeh S, et al. Reproductive and menstrual risk factors for endometriosis disease: A case-control study. Crescent J Med Biol Sci. 2019;9(1).

[12] Mollazadeh S, et al. Sexual activity during menstruation as a risk factor for endometriosis: a systematic review and meta-analysis. Int J Fertil Steril. 2023;17(1):1–6. 36617195 10.22074/IJFS.2022.541102.1207 PMC9807890

[13] Namazi M, et al. Impact of endometriosis on reproductive health: an integrative review. J Obstet Gynaecol. 2021;41(8):1183–91. 33645413 10.1080/01443615.2020.1862772

[14] Yousif AM, Abdallah WG, Mahmoud H. Implemented nursing strategy based on health promotion model for alleviating endometriosis relating symptoms. Int J Novel Res Healthc Nurs. 2019;6(3):332–44.

[15] Creswell JW, Clark VLP. Designing and conducting mixed methods research. 2007.

[16] Andres MP, Borrelli GM, Abrão MS. Endometriosis classification according to pain symptoms: can the ASRM classification be improved? Best Pract Res Clin Obstet Gynaecol. 2018;51:111–8. 30029959 10.1016/j.bpobgyn.2018.06.003

[17] Apostolopoulos NV, et al. Association between chronic pelvic pain symptoms and the presence of endometriosis. Arch Gynecol Obstet. 2016;293(2):439–45. 26329801 10.1007/s00404-015-3855-2

[18] Radhika AG, et al. A multivariate analysis of correlation between severity and duration of symptoms, patient profile and stage of endometriosis. Open J Obstet Gynecol. 2016;6(10):615.

[19] Vercellini P, et al. Association between endometriosis stage, lesion type, patient characteristics and severity of pelvic pain symptoms: a multivariate analysis of over 1000 patients. Hum Reprod. 2007;22(1):266–71. 16936305 10.1093/humrep/del339

[20] Speziale HS, Streubert HJ, Carpenter DR. Qualitative research in nursing: advancing the humanistic imperative. Lippincott Williams & Wilkins; 2011.

[21] Elo S, Kyngäs H. The qualitative content analysis adaptation. J Adv Nurs. 2008;62(1):107–15. 18352969 10.1111/j.1365-2648.2007.04569.x

[22] Guba E, Lincoln Y. The countenances of fourth generation evaluation. Description, judgment, and negotiation (mimeographed). Bloomington, Ind.. Indiana University, School of Education. Inquiry Program Area. 1985.

[23] Waltz CF, Strickland OL, Lenz ER. Measurement in nursing and health research. Berlin: Springer publishing company; 2010.

[24] Lawshe CH. A quantitative approach to content validity. Pers Psychol. 1975;28(4):563–75.

[25] DiIorio CK. Measurement in health behavior: methods for research and evaluation. John Wiley & Sons; 2006.

[26] O’Connor T, Fealy GM, Kelly M, Mc Guinness AM, Timmins F. An evaluation of a collaborative approach to the assessment of competence among nursing students of three universities in Ireland. Nurse Educ Today. 2009;29(5):493–9. 19111940 10.1016/j.nedt.2008.11.014

[27] Polit DF, Beck CT, Owen SV. Is the CVI an acceptable indicator of content validity? Appraisal and recommendations. Res Nurs Health. 2007;30(4):459–67. 17654487 10.1002/nur.20199

[28] Meyers LS, Gamst G, Guarino AJ. Applied multivariate research: design and interpretation. Sage publications; 2016.

[29] Polit DF, Beck CT. The content validity index: are you sure you know what’s being reported? Critique and recommendations. Res Nurs Health. 2006;29(5):489–97. 16977646 10.1002/nur.20147

[30] Mirghafourvand M, et al. Psychometric evaluation of the endometriosis impact questionnaire (EIQ) in an Iranian population. BMC Womens Health. 2024;24(1):135. 38378552 10.1186/s12905-024-02975-7 PMC10877831

[31] Moradi M, et al. The Endometriosis Impact Questionnaire (EIQ): a tool to measure the long-term impact of endometriosis on different aspects of women’s lives. BMC Womens Health. 2019;19(1):64. 31088434 10.1186/s12905-019-0762-x PMC6518659

[32] Namazi M, et al. Endometriosis reproductive health questionnaire (ERHQ): a self-administered questionnaire to measure the reproductive health in women with endometriosis. J Gynecol Obstet Hum Reprod. 2021;50(3):101860. 32652304 10.1016/j.jogoh.2020.101860

[33] Nourizadeh R, Mohammadi E, Simbar M. Women’s coping with an unplanned pregnancy. J Payesh. 2015;1:37–84.

[34] Tuleya LGE. Thesaurus of psychological index terms. Washington, DC: American Psychological Association; 2007.

[35] Pourafkari N. A comprehensive dictionary of psychology psychiatry: English to Persian. 2nd ed. Tehran: Farhang Moaser Publications; 2001.

[36] McEwen M, Wills EM. Theoretical basis for nursing. Lippincott Williams & Wilkins; 2017.

[37] Taleghani F, Yekta ZP, Nasrabadi AN, Käppeli S. Adjustment process in Iranian women with breast cancer. Cancer Nurs. 2008;31(3):E32–41. 18453870 10.1097/01.NCC.0000305720.98518.35

[38] Sharpe L, Curran L. Understanding the process of adjustment to illness. Soc Sci Med. 2006;62(5):1153–66. 16112783 10.1016/j.socscimed.2005.07.010

[39] Paterson B. Myth of empowerment in chronic illness. J Adv Nurs. 2001;34(5):574–81. 11380725 10.1046/j.1365-2648.2001.01786.x

[40] Boyd MA. Psychiatric nursing: contemporary practice. Lippincott Williams & Wilkins; 2008.

[41] Lee LYK, Tsang AYK, Wong KF, Lee JKL. Using the Roy adaptation model to develop an antenatal assessment instrument. Nurs Sci Q. 2011;24(4):363–9. 21975485 10.1177/0894318411419209

[42] Bayat ZS, Ghasemi M, Mohtashami J, Naderiravesh N, Baghban AA. Correlation between coping strategies and health status of burn patients after discharge at burn centers in Tehran. Nurs Midwifery Q Shaheed Beheshti Univ Med Sci. 2010;19:36–41.

